# Understanding the Epilepsy in POLG Related Disease

**DOI:** 10.3390/ijms18091845

**Published:** 2017-08-24

**Authors:** Omar Hikmat, Tom Eichele, Charalampos Tzoulis, Laurence A. Bindoff

**Affiliations:** 1 Department of Pediatrics, Haukeland University Hospital, 5021 Bergen, Norway; omah@helse-bergen.no; 2 Department of Clinical Medicine (K1), University of Bergen, 5020 Bergen, Norway; Charalampos.Tzoulis@uib.no; 3 K.G. Jebsen Center for Research on Neuropsychiatric Disorders, University of Bergen, 5009 Bergen, Norway; tom.eichele@uib.no; 4 Department of Biological and Medical Psychology, University of Bergen, 5009 Bergen, Norway; 5 Department of Neurology, Haukeland University Hospital, 5021 Bergen, Norway

**Keywords:** *POLG*, epilepsy, mitochondria, mtDNA, mechanism, occipital lobe epilepsy, status epilepticus, stroke-like episodes

## Abstract

Epilepsy is common in polymerase gamma (POLG) related disease and is associated with high morbidity and mortality. Epileptiform discharges typically affect the occipital regions initially and focal seizures, commonly evolving to bilateral convulsive seizures which are the most common seizure types in both adults and children. Our work has shown that mtDNA depletion—i.e., the quantitative loss of mtDNA—in neurones is the earliest and most important factor of the subsequent development of cellular dysfunction. Loss of mtDNA leads to loss of mitochondrial respiratory chain (MRC) components that, in turn, progressively disables energy metabolism. This critically balanced neuronal energy metabolism leads to both a chronic and continuous attrition (i.e., neurodegeneration) and it leaves the neurone unable to cope with increased demand that can trigger a potentially catastrophic cycle that results in acute focal necrosis. We believe that it is the onset of epilepsy that triggers the cascade of damage. These events can be identified in the stepwise evolution that characterizes the clinical, Electroencephalography (EEG), neuro-imaging, and neuropathology findings. Early recognition with prompt and aggressive seizure management is vital and may play a role in modifying the epileptogenic process and improving survival.

## 1. Introduction

Mitochondria are the main source of adenosine triphosphate (ATP). This is synthesized through the process of oxidative phosphorylation (OXPHOS) carried out by the MRC which sits in the inner mitochondrial membrane. The MRC comprises more than 90 protein subunits organized into five multiheteromeric complexes (termed complexes I–V). Thirteen of the MRC protein subunits are encoded by the mitochondria’s own genome (mitochondrial DNA, mtDNA), while the remaining subunits together with more than a thousand other proteins required for mitochondrial structure and function are encoded in the nuclear DNA (nDNA) [1]. Thus, mitochondrial function is under dual genomic control and depends on a constant crosstalk between the cell nucleus and mitochondria [2].

Polymerase gamma is the DNA polymerase responsible for mtDNA replication and repair [3]. Mutations in the gene encoding the catalytic subunit, *POLG* (OMIM *174763), cause secondary mtDNA damage in the form of quantitative depletion, multiple deletions, and increased load of point mutations and are considered the most common cause of inherited mitochondrial disease [2,4]. Clinically, *POLG* mutations are associated with a wide range of overlapping phenotypes that vary depending on age from devastating infantile disorders such as Alpers syndrome (OMIM # 203700) and myocerebrohepatopathy spectrum; to juvenile and adult onset myoclonic epilepsy, myopathy and sensory ataxia (MEMSA), and ataxia neuropathy spectrum (ANS); and to late onset myopathies with progressive external ophthalmoplegia (PEO) [5,6].

Epilepsy is a common manifestation of mitochondrial disorders whether caused by mutations in mtDNA or nDNA. Available data regarding the frequency of epilepsy in patients with mitochondrial disease show seizures in 35–60% of patients with biochemically confirmed mitochondrial disorders [7,8,9]. Recent studies showed that 40% of children and 23% of adults with all types of primary mitochondrial disease experienced seizures during the disease course [10,11]. Epilepsy occurs frequently in patients with *POLG* mutations [12,13,14] and a total of 128 *POLG* mutations have been linked with seizures. The majority of these patients, however, carry at least one of three common founder mutations-c.1399G > A (p.Ala467Thr), c.2243G > C (p.Trp748Ser) and c.2542G > A (p.Gly848Ser) [15]. This work focuses on the epilepsy as a feature of POLG-related disease, highlighting the spectrum of epilepsy phenotypes, clinical recognition, and treatment options. We also provide a review of our understanding of the underlying disease mechanism.

## 2. Overview of Our Mechanistic Understanding

**Figure 1:**
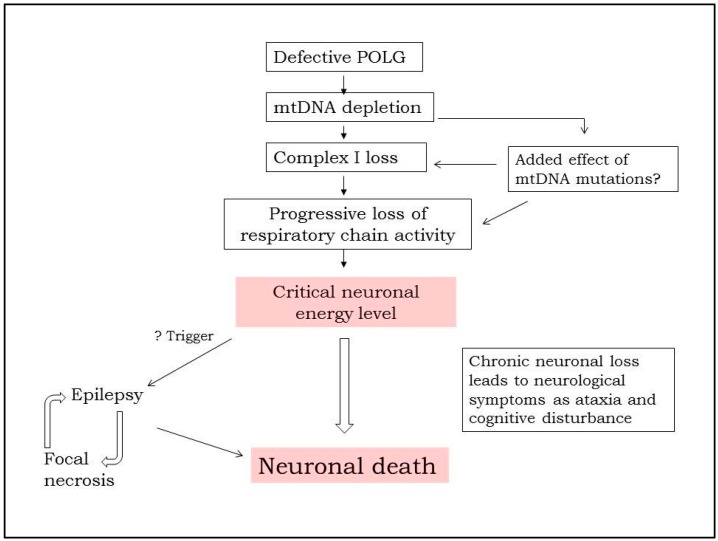
Schematic showing how *POLG* mutation can cause neuronal death and the role of epilepsy. Recessive mutations in the catalytic subunit of polymerase gamma (POLG) cause neuronal depletion of mtDNA. The level falls to ~40% and we believe that this is the threshold for neurones under which survival cannot be maintained. Depletion impairs the production of MRC components, and this particularly appears to affect complex I, which in turn leads to progressive loss of respiratory chain activity. We also find that over time (i.e., the longer a patient lives with this disease) patient neurones accumulate increasing amounts of mtDNA deletion and point mutation. Whether the greater the mtDNA mutational load has pathological consequences is unclear but possible. Based on our clinical and pathological studies, POLG related disease follows an acute on chronic course and our explanation for this is the presence of the critical neuronal energy level. Gradual loss of neurones, i.e., neurodegeneration is associated with the clinical correlates of ataxia, encephalopathy and cerebral atrophy etc. As soon as the patient develops epilepsy the picture changes dramatically .Focal necrotic lesions develop [4,17] and these are easily identified on magnetic resonance imaging (MRI), (the so-called stroke-like episodes). Such focal damage can also act as a trigger for further seizures.

Figure 1 shows a brief schematic outlining the authors’ current understanding of the mechanisms involved in POLG related epilepsy. Work performed by us and others has shown that mtDNA depletion—i.e., the quantitative loss of mtDNA—in neurones is the earliest and, in our opinion, most important factor of the subsequent development of cellular dysfunction [4,16,17]. Loss of mtDNA will lead to loss of MRC components that, in turn, progressively disables energy metabolism. This critically balanced neuronal energy metabolism can have two consequences: first, a chronic and continuous attrition (i.e., neurodegeneration) and second, it leaves the neurone unable to cope with increased demand that can trigger a potentially catastrophic cycle that results in acute focal necrosis. We believe that it is the onset of epilepsy that triggers the cascade of damage. In the following sections, we will document the findings that lead us to this mechanistic pathway starting with insights provided by the clinical features and investigations, how these were confirmed by the pathological findings and thereafter our molecular studies. Last, we will discuss the current treatment options.

## 3. Seizure Semiology and EEG Findings

Epilepsy is a common presenting feature of POLG related disease: estimates vary from approximately 50% [15] to our studies that showed 65% of patients with MEMSA [18] and more than 80% of pediatric patients have epilepsy at disease onset [19]. Focal seizures, commonly evolving into bilateral convulsive seizures, are the most common seizure types in both adult and pediatric patients, with epileptiform discharges predominantly occurring over the occipital regions, at least initially [18,20].

In juvenile- and late-onset POLG disease, seizures may be accompanied by headache and vomiting, sometimes resembling migraine with aura [13,18]. Occipital lobe features are common and may include simple flickering, colored light, visual hallucinations, scotomata, hemianopia, amaurosis, nystagmus, and ocular clonus [18]. Focal clonic or myoclonic seizures—most often involving an arm, shoulder, neck, and/or head—are present in almost all the patients and often progress into focal or/and generalized status epilepticus (SE) [15,21].

Alpers syndrome (OMIM # 203700) is one of the most common presentations of early-onset POLG disease [22] and accounts for the majority of the cases with early-onset epilepsy [19]. The causal link between *POLG* mutations and Alpers syndrome was first reported in 2004 [23]. Clinically, the disorder is characterized by refractory epilepsy, progressive encephalopathy, and liver involvement [24]. While seizure types are similar to those seen in adult-onset disease, the characteristic visual manifestations of occipital lobe involvement are less readily detected in young children. As the disease progresses, the majority of children develop myoclonic seizures and episodes of epilesia partialis continua (EPC) and/or generalized SE. Similar to the juvenile form, patients with Alpers phenotype may also present with refractory SE from which they might never recover [18,20,21,25].

Seizure activity, particularly when prolonged as in EPC or generalized SE, is closely associated with episodes of acute exacerbation previously called stroke-like episodes (SLEs). SLEs are characterized by acute or subacute neurological dysfunction and may be preceded by prodromal symptoms such as migraine-like headaches, visual disturbance, and mental changes [16]. Clinical SLEs are less often reported in children compared with adults, but radiological evidence of cortical lesions is common in both [16,19]. Since SLEs are associated with high mortality and morbidity [16] the presence of epilepsy is a negative prognostic factor: we showed that patients without epilepsy have significantly longer survival and this applied to both early- and late-onset POLG disease [17].

## 4. Epileptic Foci Correlate with Cortical Lesions on MRI

**Figure 2:**
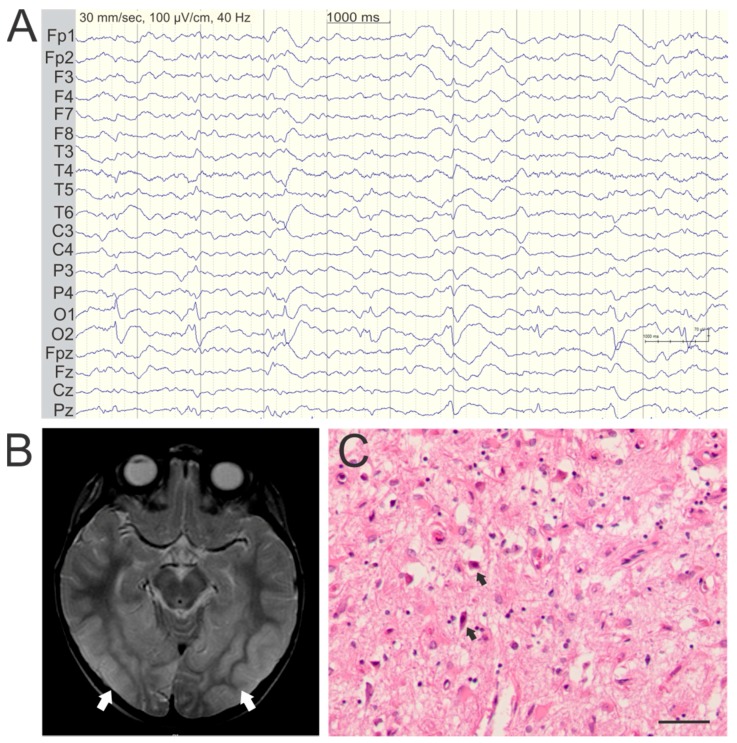
Typical EEG, imaging, and neuropathology in POLG encephalopathy. Representative findings are shown from a 41-year-old patient, homozygous for the p.W748S mutation (EEG and histology) and an 8-year-old patient compound heterozygous in trans for the p.A467T and p.G303R (MRI). (**A**) Interictal EEG recording showing periodic sharp activity around 1Hz frequency in in occipital leads O1 and O2; (**B**) Axial T2-weighted MRI shows bilateral high T2-signal consistent with cortical edema, in the occipitotemporal regions (white arrows); (**C**) Hematoxylin and eosin stained section of the medial occipital cortex showing severe vacuolation of the neuropil and neuronal loss. Arrows mark examples of eosinophilic neuronal necrosis (magnification: 200×, scalebar: 50 µm).

EEG recordings often show predominant ictal and interictal occipital epileptic activity in both adults and children with POLG disease (Figure 2A). Focal epileptic discharges may also occur in the temporal and frontal regions, and multifocal or generalized epileptic activity may occur during seizure evolution and SE [15,18]. We confirmed that prolonged seizure activity and SE can be linked with the development of cortical focal lesions (CFL) on MRI [16]. These appear as acute or subacute high T2-signal changes in the cerebral cortex, evolve rapidly over the course of days or weeks and may regress. CFL show restricted water diffusion in the acute phase, which gradually increases consistent with a transition from cytotoxic to extracellular edema [16].

CFLs occur exclusively in patients with epilepsy and, like seizure activity, predominantly affect the occipital lobes (Figure 2B), although virtually any part of the neocortex may be affected [15,16]. EEG taken during episodes of exacerbation commonly shows focal epileptic activity that correlates with the anatomical localization of the CFL. Early EEG recordings, performed within days of the SLE onset, commonly show slowing over the area of a CFL, which subsequently evolves to more distinct focal epileptiform activity [16,18]. In fact, EEG activity in some cases precedes the appearance of a CFL on MRI suggesting an upstream role in their pathogenic cascade [26]. CFLs evolve dynamically during the course of the SLE reflecting the clinical progression of the encephalopathic symptoms, and may undergo partial or complete regression, if the patient survives the episode [16,26].

Magnetic resonance spectroscopy (MRS) findings in acute CFL typically shows a prominent lactate peak and decreased *N*-acetyl aspartate concentration [16] reflecting mitochondrial dysfunction and neuronal loss, respectively. Ictal Cerebral ^18^F fluoro-deoxy-glucose positron emission tomography (FDG-PET) imaging shows increased glucose uptake in acute CFL [26].

## 5. Pathology of Cortical Lesions Is Consistent with Energy Failure

CFLs are characterized microscopically by selective, but incomplete neuronal loss, eosinophilic neuronal necrosis, vacuolation of the neuropil and diffuse astrocytosis, and microglial activation (Figure 2C). Surviving neurons with normal cytoplasmic and nuclear morphology appear evenly scattered throughout such lesions and generally show positive staining for respiratory chain complex I. The presence of surviving, complex I-replete neurons argues strongly against ischemia as a component of the damage and we have shown that there is, in fact, increased vascularization and patent vessels in the lesions [27]. This is also supported by other studies showing evidence of neovascularization and increased lesional blood flow in acute POLG lesions [4,16,17].

## 6. Molecular Disease Mechanisms Underlying POLG Related Epilepsy

We believe that the primary pathogenic event in POLG encephalopathy is neuronal energy failure due to respiratory chain dysfunction induced by mtDNA depletion. This triggers a cascade of downstream pathological changes that lead cortical damage and thus to seizure activity and ultimately neuronal death. Other mitochondrial disorders including those due mutations in the *ADCK3* gene [28], mtDNA mutations such as the m.3243A > G causing mitochondrial encephalopathy, lactate acidosis, and stroke-like episodes (MELAS, OMIM 540000) [29], and non-mitochondrial disorders such as neonatal hypoglycaemia and non-ketotic hyperglycinemia (OMIM 605899) [30] all show an occipital predilection. The common thread shared by all these conditions is energy failure, albeit caused by different mechanisms. Why this particularly affects the occipital cortex is unknown; it is among the most active regions of the brain making it perhaps more vulnerable to energy failure. However, this remains to be confirmed. Post mortem immunohistochemical studies in patients with POLG encephalopathy have shown a selective and progressive loss of complex I in neurones [4]. This finding is consistent with the previous suggestions that complex I dysfunction due to oxidative stress plays a role in the development of epilepsy in mitochondrial disease [31]. Whether arising because of an inherited defect or due to secondary oxidative stress, energy failure can drive further production of reactive oxygen species, apoptosis, and abnormal calcium homeostasis [31]. Seizures, including subclinical seizures, increase the energy demands of neurons that are already metabolically compromised thus creating a vicious circle. Intracellular energy depletion increases neuronal excitability by impairing sodium–potassium ATPase activity and decreasing the membrane potential. Impaired calcium sequestration due to mitochondrial dysfunction is also likely to occur [32,33]. Other possible contributory mechanisms include increased synaptic [34] and astrocytic glutamate release [35], which in turn lead to increased neuronal excitability and the development of epilepsy. While it is not known whether liver dysfunction contributes to epileptogenesis in POLG disease, epilepsy can develop in patients independently of clinically or biochemically detectable liver dysfunction [13].

## 7. Treatment

Epilepsy is the single most important prognostic factor associated with increased morbidity and mortality in patients with POLG disease, and we believe that early recognition and immediate, aggressive seizure treatment are crucial to improving patient survival.

The majority of the adult patients and approximately 90% of the pediatric patients [19] develop therapy resistant epilepsy [36] and almost all require high dose polytherapy. Since the most common seizure type in both in adults and pediatric patients is focal evolving to bilateral convulsive seizures, oxcarbazepine, carbamazepine, lacosamide, and perampanel are indicated and can be used in combination with a benzodiazepine such as clobazam or clonazepam. Lamotrigine, topiramate, and levetiracetam have also been used by the authors, but lamotrigine can worsen mycolonic seizures and should be used with caution. A rapid acting benzodiazepine such as midazolam could be used as an on demand medication, but has a limited role in long term treatment. In our experience, EPC is generally resistant to pharmacotherapy.

Status epilepticus should be treated quickly and aggressively. Benzodiazepines, phenytoin, levetiracetam, and occasionally phenobarbital can be used as first line treatment. If unsuccessful, generalized anesthesia using propofol or barbiturate (pentothal) should be tried, following the general guidelines for treatment of convulsive SE. Despite the concerns over the lactic acidosis associated with propofol, we have used it regularly in adult patients with POLG disease without this complication. Ketamine [37] and corticosteroids [20] have been reported to be effective in terminating SE in single cases. Sodium valproate is absolutely contraindicated in patients with POLG disease due to the risk of devastating liver disease [13], even though recovery after transient liver failure with recovery after discontinuation sodium valproate has been documented [20].

Ketogenic diet (KD) and vagus nerve stimulation (VNS) therapy are other potential non-pharmacological alternatives that can be tried in therapy resistant epilepsy. There are, however, no clinical trials confirming the benefit of either in patients with POLG disease and the authors have no direct experience of using these modalities in POLG disease to share.

Nutritional supplements such as co-enzyme Q10, carnitine, and vitamin cocktails have been widely used. However, no clear evidence showing the effect of these in improving seizures frequently and/or severity is available in the literature. Our experience is consistent with the conclusion of a previously published Cochrane systemic review of those nutritional supplements that had been tested in randomized control trials none had any demonstrable clinical efficacy [38]. Currently, there is no clear evidence showing the use of high dose coenzyme Q10, l-arginine and EPI-743 has any mentioned effect to improve the epilepsy in patients with POLG disease.
